# Seasonality of Hashimoto Thyroiditis: Infodemiology Study of Google Trends Data

**DOI:** 10.2196/38976

**Published:** 2022-09-01

**Authors:** Robert Marcec, Josip Stjepanovic, Robert Likic

**Affiliations:** 1 Department of Internal Medicine University of Zagreb School of Medicine and Clinical Hospital Centre Zagreb Zagreb Croatia

**Keywords:** Hashimoto disease, Hashimoto thyroiditis, infodemiology, search engine, Google Trends, seasonality, cosinor analysis, Google, thyroid

## Abstract

**Background:**

Hashimoto thyroiditis (HT) is an autoimmune thyroid disease and the leading cause of hypothyroidism in areas with sufficient iodine intake. The quality-of-life impact and financial burden of hypothyroidism and HT highlight the need for additional research investigating the disease etiology with the aim of revealing potential modifiable risk factors.

**Objective:**

Implementation of measures against such risk factors, once identified, has the potential to lessen the financial burden while also improving the quality of life of many individuals. Therefore, we aimed to examine the potential seasonality of HT in Europe using the Google Trends data to explore whether there is a seasonal characteristic of Google searches regarding HT, examine the potential impact of the countries’ geographic location on the potential seasonality, and identify potential modifiable risk factors for HT, thereby inspiring future research on the topic.

**Methods:**

Monthly Google Trends data on the search topic “Hashimoto thyroiditis” were retrieved in a 17-year time frame from January 2004 to December 2020 for 36 European countries. A cosinor model analysis was conducted to evaluate potential seasonality. Simple linear regression was used to estimate the potential effect of latitude and longitude on seasonal amplitude and phase of the model outputs.

**Results:**

Of 36 included European countries, significant seasonality was observed in 30 (83%) countries. Most phase peaks occurred in spring (14/30, 46.7%) and winter (8/30, 26.7%). A statistically significant effect was observed regarding the effect of geographical latitude on cosinor model amplitude (y = –3.23 + 0.13 x; *R*^2^=0.29; *P*=.002). Seasonal increases in HT search volume may therefore be a consequence of an increased incidence or higher disease activity. It is particularly interesting that in most countries, a seasonal peak occurred in spring and winter months; when viewed in the context of the statistically significant impact of geographical latitude on seasonality amplitude, this may indicate the potential role of vitamin D levels in the seasonality of HT.

**Conclusions:**

Significant seasonality of HT Google Trends search volume was observed in our study, with seasonal peaks in most countries occurring in spring and winter and with a significant impact of latitude on seasonality amplitude. Further studies on the topic of seasonality in HT and factors impacting it are required.

## Introduction

Hypothyroidism is a growing global public health problem affecting approximately 5% of the general global population [1]. The leading cause of hypothyroidism, in areas with sufficient iodine intake, is Hashimoto thyroiditis (HT)—an autoimmune thyroid disease, with an incidence of 0.3-0.5 of 1000 population per year [2]. HT can present with a long list of both local and systematic symptoms such as dyspnea, dysphagia, dysphonia, constipation, coronary artery disease, bradycardia, anemia, memory loss, depression, bile colic, and dry and thickened skin [3]. The etiology of HT is still insufficiently clarified, but it is known to be associated with genetic factors, environmental triggers, and epigenetic influences [4]. Although some predisposing factors, such as stress, age, and gender, are recognized in the pathogenesis [5], much about HT still remains unknown and warrants further investigation. Namely, hypothyroidism represents a significant global, financial, and quality-of-life burden [1]. For example, hypothyroidism-related medical costs in the United States are estimated to range from US $460 to US $2555 per patient per year, patients with hypothyroid reporting significantly higher work absenteeism and significant long- and short-term disability costs, resulting in further direct and indirect costs [6]. The high quality-of-life and financial burden of hypothyroidism and HT emphasize the need of additional research investigating the disease etiology to reveal potential modifiable risk factors. Implementing measures against such risk factors has the potential to lessen the financial burden while also improving the quality of life of many individuals. Various environmental and seasonal factors, such as insolation and UV exposure, seasonal incidence of infectious diseases, or seasonal changes in human behavior, may potentially play a role in the disease’s etiology. Vitamin D is a particularly interesting seasonal factor implicated in the pathophysiology of numerous diseases. Studies about vitamin D alteration by climate variation [7,8] along with studies about vitamin D deficiency in the pathogenesis of many autoimmune diseases, such as multiple sclerosis [7], diabetes, and also cancers, [9] have been conducted. A recently published systematic review on the topic of the association between vitamin D deficiency and autoimmune thyroid disorders found out that most of the studies included in the review supported the association between low vitamin D levels and the occurrence of autoimmune thyroid diseases, in particular HT and Graves disease, but the authors highlighted the need for further randomized long-term follow-up studies to confirm the potential causal link and potential role of vitamin D supplementation [10]. Unfortunately, such studies are costly and difficult to conduct; therefore, until such trials are performed, other sources of information may serve to narrow the evidence gap. One such area of science, which may help to provide further evidence regarding potential risk factors for HT, is infodemiology (short for information epidemiology); infodemiology studies health-related user data available on the internet with goals of improving public health, reducing the impact of web-based misinformation, and narrowing the existing knowledge gaps [11]. In recent years, the increase in internet penetration and usage as well as the number of web-based social platforms have provided a rich source of user-related health information. Therefore, multiple internet services and platforms have been used in infodemiology studies in recent years with various relevant topics being explored. Multiple studies used the data retrieved from Twitter to explore the sentiment and analyze conversation themes regarding COVID-19 vaccines, with goals of providing information relevant to fighting vaccine hesitancy [12,13], while other studies explored the potential role of Instagram in raising awareness of skin cancer [14] or analyzed the platform’s contents regarding vaping [15]. Apart from social media platforms, internet search engines represent a particularly interesting source of health-related data. Changes in internet search trends have been found to potentially reflect the general public’s interest regarding medical-related topics [16], and disease-specific internet searches tend to increase in parallel with the increase in the specific burden of disease [17,18]. Worldwide, Google is the most widely used search engine, and it also provides a freely accessible tool set called Google Trends (GT) through which one can easily conduct an analysis of search trends. With GT data being accessible in real time, the issue of lingering survey methods becomes obsolete, as the data are available practically instantly. Another major advantage of GT is the fact that it enables obtaining information that would be difficult, costly, or even impossible to obtain with conventional methods, such as the estimated population sizes and geospatial distributions of marginalized populations (eg, LGBTQ+), which is important for public health planning [19]. In addition, GT makes delicate topics and disease research, such as HIV, suicide rates, and mental illnesses, more easily doable as web searches are executed anonymously [20]. Thus, GT has so far been used in a number of studies investigating a broad range of medical topics, and based on the GT data, seasonal patterns have been proposed for various diseases ranging from psoriasis [21], gout [22], bruxism [23], and cellulitis [24] to major mental disorders [25].

Therefore, the aim of this study was to explore the potential seasonal pattern of Google searches regarding HT in European countries in order to guide future real-world studies on this topic.

## Methods

### Research Questions

Our study examined the seasonality of HT Google-related searches in Europe using GT data, with the goal to explore whether there was a seasonal characteristics of Google searches regarding HT, examine the potential impact of the countries’ geographic location on the potential seasonality, and identify possible modifiable risk factors for HT, thereby inspiring future research on the topic.

### Data Retrieval

We conducted our GT data retrieval throughout May 2021, and using all the search categories, we queried GT using the search topic “Hashimoto thyroiditis.” The data were retrieved for 38 European countries, covering a 17-year period of time, from January 2004 to December 2020. A total of 36 European countries were included in the analysis, while Kosovo and Moldova were excluded due to an extremely small search volume. As we investigated the potential impact of seasonality on Google searches, the widest available search time frame of 17 years was used. A specific search topic and no search category restrictions were used to potentially capture the interest of the population of multiple European countries, as wide as possible. The search topic approach was used as it included alternative spellings and translations in other languages; this approach, when comparing European countries using different languages in their Google searches, provided a simple and uniform way to extract the data for each country. The methods were reported following suggestions from a systematic review article on the topic of the use of GT in health care research [18].

### Data Analysis

GT search data were expressed as relative search volume (RSV) normalized to range from 0% to 100% for the set search time frame and geographical location. Seasonality was assessed using a cosinor regression model from the R programming language “seasons” package. The model fits a sinusoid to the monthly input data, and its outputs include the sinusoid’s amplitude, the phase corresponding to the sinusoidal peak, and 2 *P* values, the smaller of which is being reported in Table 1. A *P* value of less than .025 was considered statistically significant. More information on the cosinor model can be found in studies by Cornelissen [26], Mei et al [27], and Wu et al [21]. Months with 0 RSV have been encoded as NA, allowed by the model.

Seasons have been defined as spring (March, April, and May), summer (June, July, and August), autumn (September, October, and November), and winter (December, January, and February).

To evaluate the potential influence of the countries’ latitudes and longitudes, the weighted population center coordinates for each country were retrieved from the Baylor University population resource [28] except for Serbia and Montenegro, where the coordinates of the capital cities were used instead, as the weighted population center coordinates for the 2 countries were not reported in the population resource.

Simple linear regression was conducted to evaluate the potential effect of latitude and longitude on seasonal amplitude and phase of the cosinor model output for each country.

All statistical analyses and data visualizations were done in R programming language (version 4.0.5; R Core Team).

**Table 1:** Cosinor model analysis results regarding country seasonality, amplitude, phase, and cosinor *P* values. Phase corresponds to month. Number of observations column correspond to the number of months for each country with a Google Trends relative search volume. Latitude and longitude values are used in the simple linear regression.

Country	Seasonality	Amplitude	Phase	Phase season	*P* value	Number of observations	Latitude	Longitude
Albania	Yes	10.72	7.4	Summer	<.001	50	41.174529494701	19.929275580053
Austria	Yes	2.24	7	Summer	<.001	191	47.765386201318	14.645625300333
Belarus	Yes	1.67	9.3	Autumn	<.001	115	53.531624124024	27.847175354981
Belgium	Yes	2.17	3.3	Spring	.002	175	50.844005826061	4.4332869095216
Bosnia and Herzegovina	No	—^a^	—	No seasonality	.07	118	44.160791721547	17.753208075376
Bulgaria	Yes	2.21	7.3	Summer	<.001	180	42.754116369708	25.083976957381
Croatia	Yes	2.06	1	Winter	.001	152	45.317637428417	16.262950671815
Czech Republic	Yes	2.1	7.5	Summer	<.001	113	49.821456149539	15.617527756779
Denmark	Yes	2.01	2.9	Winter	.007	144	55.853326754724	10.856715208377
Estonia	Yes	5.63	8.7	Summer	<.001	48	58.957945858648	25.572740786761
Finland	Yes	6.19	2.3	Winter	<.001	126	61.755732589277	24.98467066628
France	Yes	2.58	4.7	Spring	<.001	202	47.143228746162	2.6764463428893
Germany	Yes	3.62	5.2	Spring	<.001	204	50.855573924694	9.6963409646128
Greece	Yes	1.49	5.9	Spring	.02	173	38.686808689502	23.323965300494
Hungary	Yes	1.75	4.5	Spring	<.001	146	47.288770753717	19.388772968949
Iceland	Yes	7.21	5.1	Spring	<.001	54	64.372216876845	–21.045029641756
Ireland	No	—	—	No seasonality	.57	121	53.111585555903	–7.4282382442794
Italy	Yes	3.43	4.3	Spring	<.001	203	42.870086858764	12.12890612484
Latvia	No	—	—	No seasonality	.24	96	56.831191706188	24.496056054831
Lithuania	Yes	3.63	8.4	Summer	<.001	63	55.223194780885	23.887086150639
Luxembourg	Yes	5.58	3.6	Spring	<.001	101	49.643734947502	6.0837996175026
Macedonia	Yes	4.66	2.4	Winter	<.001	79	41.742844591767	21.554126089671
Montenegro	Yes	3.96	3.3	Spring	<.001	73	42.442574	19.268646
Netherlands	Yes	2.61	2.3	Winter	.001	193	52.072871145825	5.2875541627667
Norway	Yes	3.77	5.9	Spring	<.001	123	61.128336570352	9.9468336009803
Poland	Yes	2.56	2.5	Winter	.005	196	51.707976823759	19.308388806995
Portugal	No	—	—	No seasonality	.11	157	39.74693753116	–9.1672490596965
Republic of Serbia	Yes	1.46	3.5	Spring	.02	150	44.787197	20.457273
Romania	Yes	1.93	10.4	Autumn	.001	161	45.692835166704	25.283411622442
Slovakia	Yes	3.2	12.8	Winter	<.001	98	48.662536038829	19.164300350224
Slovenia	Yes	2.5	3.9	Spring	<.001	111	46.169295822703	14.89236963709
Spain	Yes	2.01	5.2	Spring	.008	191	39.720397339383	–3.2923251997811
Sweden	No	—	—	No seasonality	.04	165	58.913317696441	15.528746561364
Switzerland	Yes	2.68	4.2	Spring	<.001	185	47.025712614417	7.9586515381984
Ukraine	No	—	—	No seasonality	.38	153	48.808076188342	31.766935926448
United Kingdom	Yes	2.03	2.4	Winter	<.001	196	52.745166767654	–1.6847761296012

^a^Not applicable.

### Ethical Considerations

The ethical committee of the University of Zagreb School of Medicine exempted this study from review.

## Results

Cosinor model results on the seasonality of the search term “Hashimoto thyroiditis” in 36 European countries can be seen in Multimedia Appendix 1 and Table 1. Boxplot graphs in Multimedia Appendix 1 represent the monthly GT RSV for each country along with the sinusoid resulting from the cosinor model.

Of the 36 included European countries, significant seasonality was observed in 30 (83%) countries, with a mean amplitude of 3.3 (SD 2.0; median=2.6) and a mean phase value of 5.24 (SD 2.8; median=4.6).

**Figure 1:**
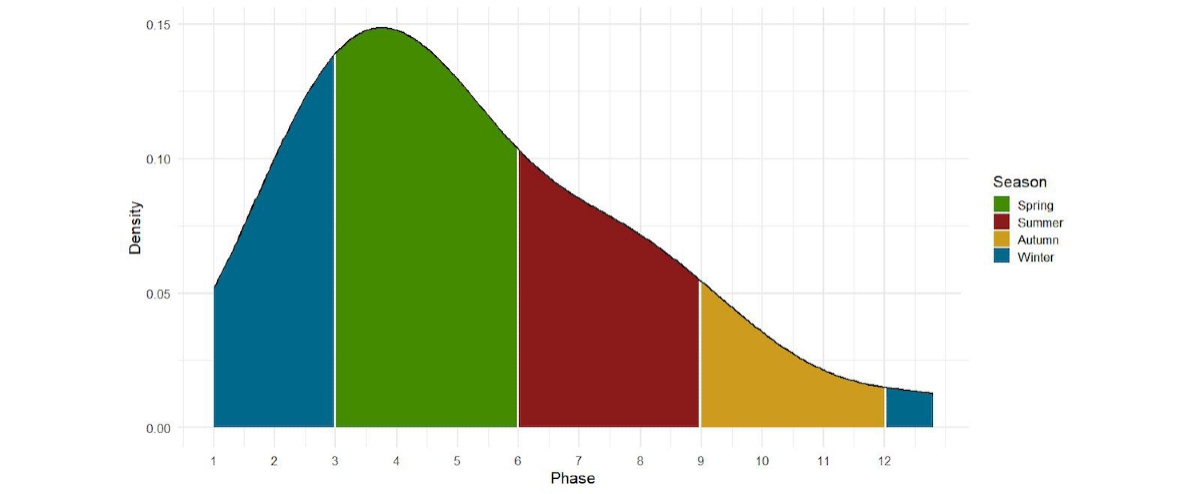
Density plot showing the distribution of phase months.

**Figure 2:**
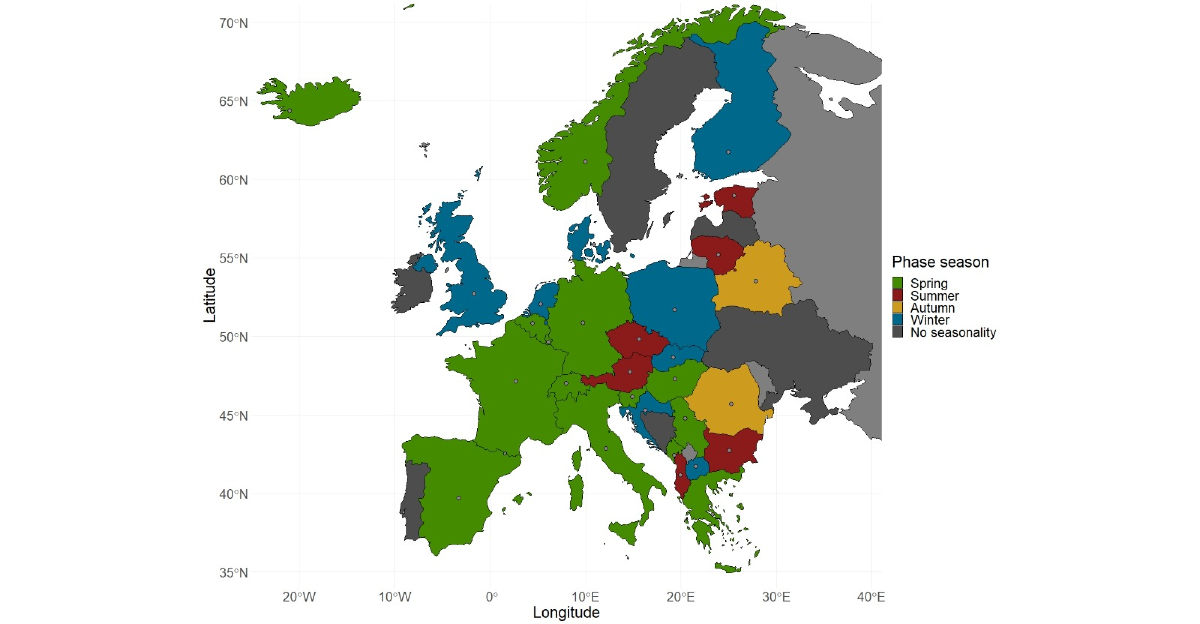
Map of Europe colored by season of phase. Points represent weighted population centers for each country, except Serbia and Montenegro, where coordinates of capital cities were used.

Distribution of the phase months can be seen in Figure 1; most of the phase peaks occurred during spring (14/30, 46.7%), winter (8/30, 26.7%), and summer (6/30, 20%), while the least phase peaks were in autumn (2/30, 6.7%). Geographical distribution of phase seasons is shown in Figure 2.

**Figure 3:**
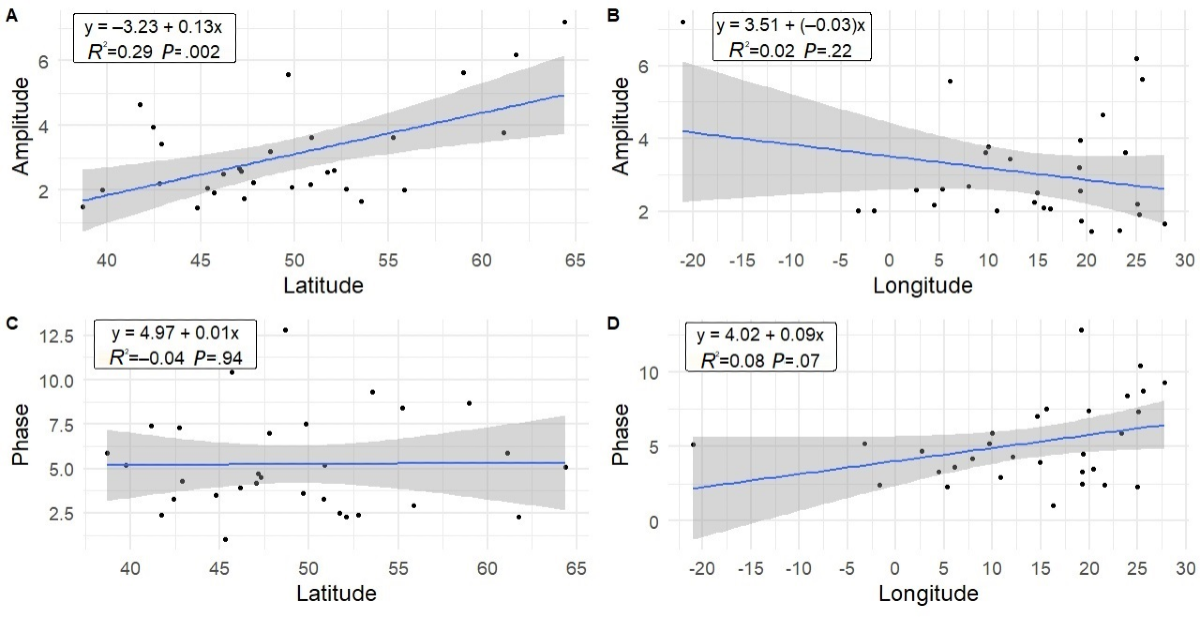
Graphs showing simple linear regression results of the impact of latitude and longitude on amplitude and phase.

Simple linear regression results of the effect of latitude and longitude on seasonal amplitude and phase of the cosinor model are demonstrated in Figure 3. A statistically significant effect was observed regarding the effect of latitude on seasonality amplitude (y = –3.23 + 0.13 x; *R*^2^=0.29; *P*=.002). No statistically significant effects were observed regarding the effect of latitude on phase month (*P*=.22), longitude on amplitude (*P*=.94), or longitude on phase month (*P*=.07). The amplitude value of Albania was excluded from the linear regression models as it was identified as an extreme outlier, most likely due to low quantity of the monthly RSV data.

## Discussion

### Principal Findings

The results of our study demonstrate statistically significant seasonality in HT-related RSV across Europe, with most seasonal peaks occurring in spring and winter months (22/30, 73.3%). Additionally, we have also observed a statistically significant impact of geographical latitude on seasonal amplitude.

### Comparison With Prior Work

A study exploring internet searches patterns regarding hypothyroidism found similar results, with more hypothyroidism-related internet searches occurring during spring globally [29]. Seasonal increases in RSV for HT may be a consequence of increased incidence or higher disease activity. Namely, the volume of disease-related internet searches is known to correlate with patients’ desire to gather more information before an appointment and to supplement information provided by the physician [30]. It is particularly interesting that in most countries, a seasonal peak occurred in spring and winter months; when viewed in the context of the statistically significant impact of geographical latitude on seasonality amplitude, this may indicate that vitamin D levels could play a role in the seasonality of HT. Significant discrepancies in vitamin D levels between northern and southern European countries have been observed, with people in northern European countries having lower levels of vitamin D, which can be associated with less sun exposure, geographical latitude, and solar zenith angle [31]. People living in higher-latitude countries receive lower yearly amounts of sunlight, which leads to a predisposition for developing vitamin D deficiency. Studies have shown that shorter days and insufficient sunlight exposure at latitudes above 40 degrees North lead to poor vitamin D synthesis in the skin [7]. Furthermore, seasonal changes in serum vitamin D levels have been implicated in the seasonality and outcomes of infectious disease [32]. Vitamin D levels are also known to have seasonal fluctuations with the lowest serum levels occurring in the late winter and early spring months [33], which seems to correlate well with the seasonal increase in the RSVs of HT in most countries. Research by Kim [34] and Jamka et al [35] highlighted the importance of vitamin D in the pathogenesis of HT. Kim’s [34] cross-sectional study showed significantly higher prevalence of vitamin D insufficiency in patients with autoimmune thyroid disease, while Jamka et al [35] demonstrated a presumed vitamin D effect on reduction in the levels of thyroid peroxidase antibodies, which has an important role in HT pathogenesis [36]. Multiple studies have demonstrated beneficial effects of vitamin D supplementation on autoimmune diseases, but most notable is the recently published study in the *BMJ*, which found that vitamin D supplementation (2000 IU/day) could reduce autoimmune disease rate by 22% [37]. Future research should focus on the association between vitamin D serum seasonal changes as a potential trigger of HT and vitamin D supplementation during winter and early spring months, which may prove to be an easily implemented public health measure to decrease the burden of HT.

### Limitations

It is important to consider some of the possible limitations of this study. First, selection bias might be present, as this study only used data pertaining to the population with internet access and those who used Google instead of other search engines. Second, medical-related searches may be performed by anyone interested in a particular medical topic and not only patients. Third, deeper analysis of individual users could not be performed due to the limitations of the available data set. Finally, the impact of potential confounding factors, such as academic cycling (ie, higher search volumes in spring during college exams), could not be excluded.

### Conclusions

Significant seasonality of GT search volume for HT was observed in our study, with seasonal peaks in most European countries occurring during spring and winter. A significant impact of latitude on seasonality amplitude was also demonstrated. Additional studies on the topic of seasonality in HT and factors impacting it are required. If vitamin D deficiency is unequivocally proven as a contributing factor in the development of HT, vitamin D supplementation during winter and early spring months might be an easily implemented public health measure aimed at decreasing the burden of this disease.

## Appendix

Multimedia Appendix 1 Boxplot graphs showing monthly Google Trends data with cosinor model output. Y-axis represents relative search volume.

## Abbreviations

GT: Google Trends
HT: Hashimoto thyroiditis
RSV: relative search volume
