# Supplementary material for: Seasonality of Hashimoto Thyroiditis: Infodemiology Study of Google Trends Data
Source: JMIR Bioinform Biotechnol. 2022 Sep 1;3(1):e38976. doi: 10.2196/38976 (PMC11135219; doi:10.2196/38976)

**Multimedia Appendix 1**

Boxplot graphs showing monthly Google Trends data with cosinor model output. Y-axis represents relative search volume.


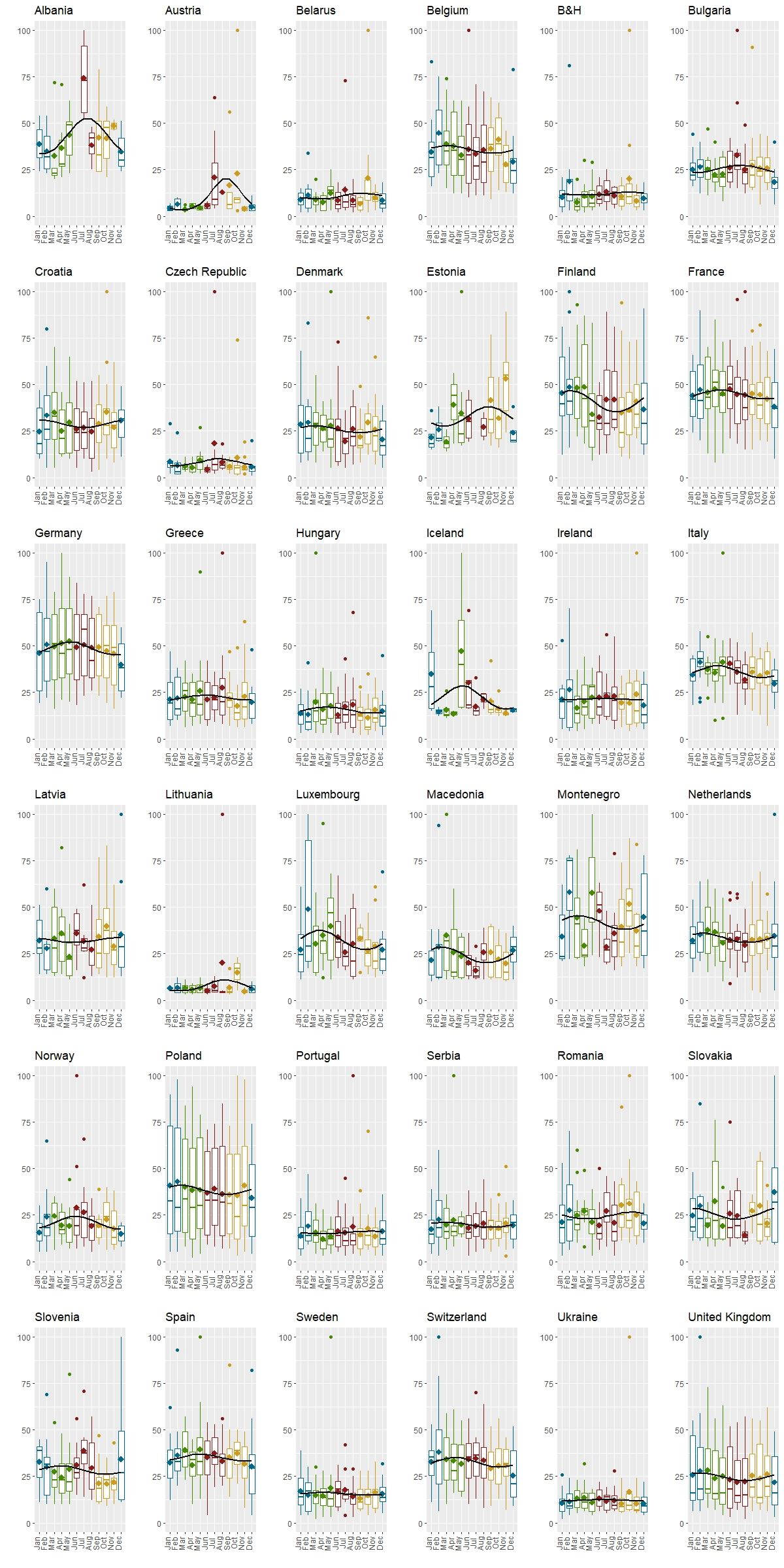

Supplement: Multimedia Appendix 1 [file bioinform_v3i1e38976_app1.docx]
